# A systematic literature review of breastfeeding interventions among Black populations using the RE-AIM framework

**DOI:** 10.1186/s13006-022-00527-z

**Published:** 2022-12-17

**Authors:** Emiliane Lemos Pereira, Paul A. Estabrooks, Alejandro Arjona, Wyconda Cotton-Curtis, Judith C. P. Lin, Carrie L. Saetermoe, Kacie C. A. Blackman

**Affiliations:** 1 Department of Health Promotions, University of Nebraska Medical Center, Omaha, USA; 2 Department of Health & Kinesiology, University of Utah, Salt Lake City, USA; 3 Department of Family & Consumer Sciences, California State University Northridge, Northridge, USA; 4 Department of Health Sciences, California State University Northridge, Northridge, USA; 5 The Health Equity Research and Education Center, California State University Northridge, Northridge, USA

**Keywords:** Breastfeeding initiation, Breastfeeding duration, Breastfeeding continuation, African Americans / Black

## Abstract

**Background:**

Lactation support resources are less likely to be located in close proximity to where Black families live and there is a systemic racist health care belief that Black women prefer bottle feeding (with infant formula) over breastfeeding. Together, these lead to lower reported breastfeeding rates of Black babies compared to other racial / ethnic groups. It is imperative to have a deeper understanding of the cultural aspects as well as the underlying limitations that prevent Black women / persons from being supported to breastfeed. There is a need to know how effective breastfeeding interventions are in reaching the intended population; how well they work in promoting breastfeeding initiation and continuation; and how successful they are when implemented at the setting and staff level. The purpose of this investigation was to establish the level of internal and external validity that was reported by breastfeeding intervention studies among Black communities.

**Methods:**

Studies on breastfeeding interventions on Black people that were published between the years 1990 and 2019 were carefully examined through PubMed, EBSCOhost, Web of Science, and OneSearch. A total of 31 studies fulfilled the requirements to be included for this evaluation. In order to extract the information from the articles, the reach, effectiveness, adoption, implementation, and maintenance (RE-AIM) framework extraction tool was utilized.

**Results:**

On average, the proportion of studies that reported across reach, effectiveness, adoption, implementation, and maintenance indicators was 54, 35, 19, 48, and 9%, respectively. Across core RE-AIM indicators only sample size (100%) and breastfeeding outcomes (90%) were reported consistently. External validity indicators related to representativeness of participants (16%) and sites (3%) were rarely reported. Similarly, adherence to intervention protocol, and indicator of internal validity, was reported in a small proportion of articles (19%).

**Conclusion:**

This body of literature under-reported on aspects associated to both internal and external validity across all RE-AIM domains. The reporting of the individual level of representativeness; the setting level of representativeness; the intervention’s adherence to the protocol; the expenses; and the factors of sustainability would benefit from improvement in future research.

**Supplementary Information:**

The online version contains supplementary material available at 10.1186/s13006-022-00527-z.

## Background

The US National breastfeeding recommendations are that infants need to be exclusively breastfed for at least the first 6 months of life [1]. However, the US national rates show that only 58% of the infants born in 2017 were breastfeeding until 6 months [2]. Furthermore, breastfeeding rates for Black infants are disproportionately lower with only 17% of Black infants exclusively breastfed until the age of 6 months (vs. 30% of white infants) in the US [3]. Moreover, there are many factors that contribute to these rates such as access and affordability to quality breastfeeding support [4], racially targeted marketing of infant formula [5], and employment inequities in the US (e.g., lack of access to paid leave) [6]. Compared to White, Asian, and Hispanic women, Black women received fewer weeks of full-pay equivalent pay during their parental leave and had significantly less access to paid leave through their employers or through government programs. These inequitable differences can exacerbate structural racism which impacts racial disparities in breastfeeding rates. The cost of not breastfeeding leads to lasting health and economic adverse effects (e.g., high medical costs to common childhood illness, and household formula costs) [7]. In the event that breastfeeding can happen as planned, these aforementioned adverse effects are reduced which results in minimal treatment costs for common childhood illnesses [7].

Though breastfeeding initiation has increased, there are still some factors that impact breastfeeding duration long-term such as marital status, age, education, social economic status, racism in the workplace and structural barriers of women and work lifestyle [8]. In a scoping review, Robinson et al. [9] demonstrated that Black women experience racism, bias, and discrimination (e.g., assumption that Black women do not breastfeed, less breastfeeding referrals) which affect breastfeeding care, support, and outcomes.

Since breastfeeding rates are lowest among Black people, Eurocentric-informed interventions may not be effective and therefore culturally-informed interventions are necessary to support these families. Past literature reviews on breastfeeding promotion interventions have demonstrated some effectiveness with providing breastfeeding support for Black people [10–12] . These studies demonstrate the critical importance of including cultural and historical components in breastfeeding treatments targeted at Black lactating women for them to be effective. However, these reviews have not reported on the degree to which interventions provide information on the potential reach, implementation quality, or the potential for sustained implementation — key indicators for both internal and external validity. This includes a lack of reporting on culturally-informed interventions and potential implementation strategies that can be used to navigate established barriers such as institutional racism and shorter maternity leave when compared with other races, and lower wage jobs [6]. These factors collectively are potential contributors to the likelihood of breastfeeding initiation, duration, and continuation [12]. The purpose of this paper was to determine the degree to which breastfeeding interventions among Black populations reported on internal and external validity — specifically: intervention reach into the intended population; effectiveness in supporting breastfeeding initiation and continuation, setting and staff level adoption of breastfeeding interventions; implementation as intended; and potential for intervention maintenance at the organizational level [13]. This assessment can provide guidance on summarizing findings to inform research to practice translation and identify methodological gaps related to research design and evaluation.

## Methods

The RE-AIM framework, a model developed to address the issue of the external validity and translation of the research into practice was used in this paper [13] (see Additional file 1). This framework uses five different dimensions: reach (R), effectiveness (E), adoption (A), implementation (I), and maintenance (M) with organizational and individual levels embedded on their dimensions [14].

### Study design

In July 2020, a systematic review was conducted.

### Systematic review protocol

The first methodology that was used included identifying five potentially relevant literature review articles [8–12]. These review articles were identified using the search terms below and including “literature review”, “review”, and “systematic review”. A reverse citation (i.e., looking at the works cited by the author) was conducted on these five articles which helped with identifying more articles. The next steps of the methods and reporting were developed and conducted with systematic methodology using MeSH terms and text words and a librarian checked the strategy. The Preferred Reporting Items for Systematic Reviews and Meta-Analyses (PRISMA) checklist was used as a guide for reporting [15].

### Data sources

Across four databases, the search strategies utilized were: PubMed, EBSCOhost, Web of Science and California State University Northridge OneSearch.

### Search strategy

See Additional file 2 for the search strategy. Search terms included “breastfeeding interventions”, “African American mothers”, “breastfeeding initiation” and “breastfeeding duration”.

### Inclusion and exclusion criteria

The eligibility of studies for review was assessed using the following inclusion criteria: (a) population of Black, African American, Latino / a (Black), Latino / a (African), Afro-Latino / a, Afro Latino / a, Afro-Caribbean, Afro Caribbean, (b) articles in English, (c) studies that conducted experimental or quasi-experimental designs, (d) studies that included a comparator, (e) at least two data collection points (pre- and post-collection and maintenance) and (f) included at least one of these primary outcomes: breastfeeding initiation, breastfeeding continuation or breastfeeding duration. The set of criteria is shown in Table 1. All systematic reviews, theses, dissertations, and letters to the editor were excluded.

**Table 1:** Inclusion criteria

Data type	Inclusion criteria
Participants	Black, African American, Latino/a (black), Latino/a (African), Afro-Latino/a, Afro Latino/a, Afro-Caribbean, Afro Caribbean
Language	English
Study design	Used experimental or quasi-experimental design
Control condition	Any comparator including active control, inactive control, or pre- and post-measure
Assessments	Must include at least two data collection points (pre and post assessment)
Primary outcome(s) (at least one of these outcomes)	Breastfeeding initiation, continuation, and duration

### Data management

The references were organized using Zotero, an open-source software reference manager. Relevant studies were exported from databases to Zotero.

### Data extraction

Research assistants (*n* = 8) completed training on the RE-AIM framework and data extraction process provided by the senior author (KB), an expert in the application of the framework [16, 17]. The inclusion criteria used in this literature review can be found in Table 1. Articles that did not meet the inclusion criteria based on the title, abstract, or full text review were excluded.

A number of studies have used a RE-AIM data extraction tool, available on the RE-AIM website over the past 20 years. The tool includes core components from an initial definition of RE-AIM dimensions: (a) the number, proportion, and representativeness of participants (reach); (b) changes in the primary outcome, quality of life, and unintended negative consequences (effectiveness); (c) the number, proportion, and representativeness of settings and staff that agree to deliver the intervention (adoption); (d) the degree to which the intervention is delivered as intended and related cost (implementation); and (e) sustained improvements in the primary outcome post-intervention, attrition at follow-up, and description of program continuation post-study (maintenance) [18, 19]. Additional indicators have been developed and used more recently that provide a broader set of indicators across RE-AIM dimensions [14, 20, 21]. Of note, we also included a field to track the degree to which the articles addressed cultural components relevant to the Black community (see Additional file 3). All articles were reviewed, and data were extracted based on the RE-AIM indicators. All discrepancies were resolved through co-author discussions.

### Data analysis

The findings for reach, effectiveness, adoption, implementation, and maintenance indicators are reported as a percentage across studies for each indicator, and summary indicators for both core indicators and across all assessed indicators.

## Results

### Search

**Fig. 1:**
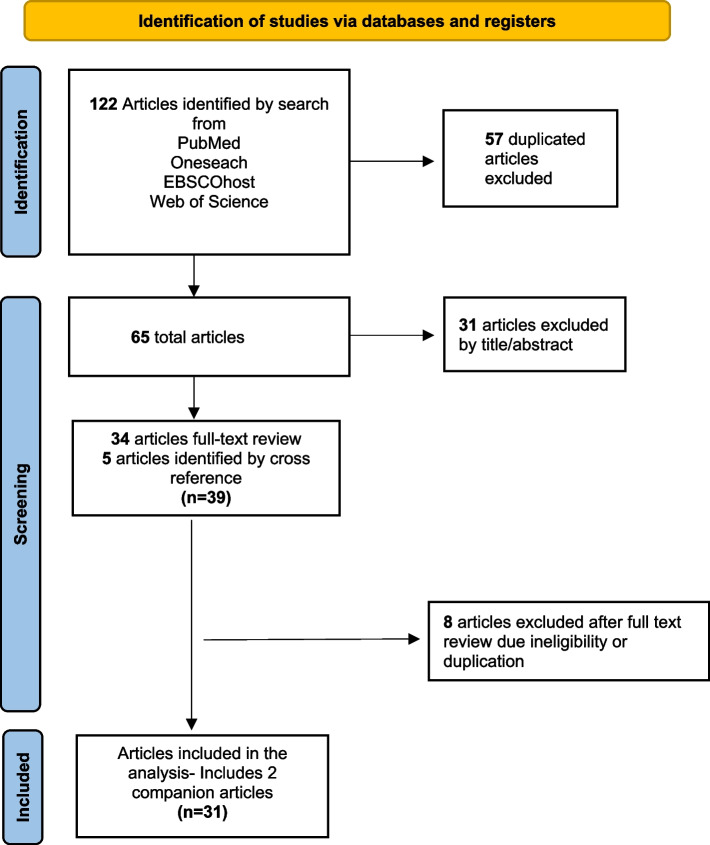
PRISMA Flow Diagram

The search retrieved 122 articles with publication years spanning 1990 to 2020. After removing duplicates, 57 articles remained for screening at the title and abstract stage. The remaining 34 articles were reviewed at full-text stage with the addition of five articles identified by cross-referencing. Eight articles were excluded after full-text review due to ineligibility or duplication. Eligible papers (*n* = 31) represented 29 unique intervention studies included in this review. See Fig. 1 for details.

### Overall summary

Across the 31 articles, inter-rater reliability was over 80%. Table 2 provides an overview of the results. Nineteen randomized controlled trials were included. Publication dates ranged from 1990 to 2019 for the 31 eligible articles. All included interventions took place in the United States with study outcomes of breastfeeding initiation, continuation, and duration. Study settings included prenatal clinics, Women, Infants and Children (WIC) clinics, hospitals, community health centers and participants’ homes. Quantitative methods were used in 29 studies and individual level of analysis was used in 16 studies. Interventions’ study duration of counseling interventions ranged from 5 min to 3 years and information regarding program duration was reported on 27 studies.

**Table 2:** RE-AIM indicators with the number and percent of studies (*N* = 31) reporting each indicator

Individual (Participant)-Level	# Studies Reporting	% Studies Reporting
Reach		
*Participation rate*	12	39
*Representativeness*	5	16
*Sample size*	31	100
Description of intended audience	31	100
Inclusion criteria	27	87
Exclusion criteria	21	68
Number of eligible exposed to recruitment	16	52
Participant attendance/completion	10	32
Method to identify intended audience	24	77
Description of recruitment methods used	20	65
Used qualitative methods to describe reach	2	7
Core indicator reporting		52
Overall reach reporting		54
Efficacy/effectiveness		
*Breastfeeding outcomes*	28	90
*Quality of life*	0	0
*Unintended negative consequences*	1	3
Attrition	20	65
Type of analysis	16	52
Cost effectiveness	2	7
Used qualitative methods to describe effectiveness	9	29
Core indicator reporting		31
Overall efficacy/effectiveness reporting		35
Maintenance		
*Individual level maintenance*	5	16
*Attrition at follow-up*	6	19
Use of qualitative methods to describe individual level maintenance	2	7
Core indicator reporting		18
Overall maintenance reporting		14
Setting Level		
Adoption		
*Number of participating sites*	18	58
*Site participation rate*	5	16
*Site representativeness*	1	3
*Number of staff that agreed to deliver intervention*	5	16
*Staff participation rate*	1	3
*Staff representativeness*	0	0
Description of intended settings	11	36
Description of intervention locations	9	29
Method to identify settings	6	19
Site level inclusion/exclusion criteria	2	7
Number of eligible and invited sites	8	26
Method to identify delivery staff	6	19
Number of staff eligible and invited to participate	0	0
Staff level inclusion/exclusion criteria	2	7
Level of delivery staff expertise	21	68
Characteristics of staff	5	16
Cost of adoption	1	3
Used qualitative methods to describe adoption	3	10
Core indicator reporting		16
Overall adoption reporting		19
Implementation		
*Adherence to intervention protocol*	6	19
*Measure of cost*	3	10
Intervention description	30	98
Intervention duration	27	87
Number of intervention contacts	26	84
Timing of intervention contacts	25	81
Description of theory or principles used to develop the intervention	10	32
Consistency of implementation	3	10
Use of qualitative methods to describe implementation	3	10
Core indicator reporting		15
Overall implementation reporting		48
Maintenance		
*Description of program continuation post study*	4	13
Description of integration into delivery system	1	3
Use of qualitative methods to describe organizational level maintenance	0	0
Ongoing maintenance costs	1	3
Core indicator reporting		13
Overall maintenance reporting		5

Italicized items reflect core indicators based on the RE-AIM framework

### Reach

The proportion of studies that reported across reach indicators was 54% overall, and 52% for core indicators. All the studies reported on sample size and 39% reported on participation rate, but only 16% reported on representativeness. Participation rates ranged from 28 to 89%. Sample sizes ranged from 25 to 5886 participants (median:196). Cost of recruitment was only reported by two studies (6%) and ranged from US$15 to US$20 dollars.

### Efficacy / effectiveness

The proportion of studies that reported across effectiveness indicators was 35% overall, and 31% for core indicators. Most studies (90%) showed improvements on breastfeeding initiation (reported *p*-value, confidence intervals, percentages) and / or duration on Black population. Some studies employed in-person contacts, counselors, peer counselor (mother, family members), doulas, and specialized prenatal care; these had an effective impact on interventions success. Of the nine studies that reported implementing at least one cultural component (e.g., focus group with target population to inform study design), six reported statistically significant breastfeeding outcomes. Of the 11 studies that reported statistically significant breastfeeding outcomes, five did not implement at least one cultural component. Only one study (3%) reported unintended negative consequences. None of the studies reported quality of life. Of the two studies that reported cost effectiveness, one study reported no significant difference in raw cost of prenatal care, and the other study showed that the intervention was potentially cost effective, however, did not provide clear data on cost savings.

### Adoption

The overall proportion of studies that reported across adoption indicators was 19%, and was 16% for core indicators. The number of participating sites was reported in 18 studies (58%), but site level participation rate (16%) and site level representativeness (3%) were not reported often. Similarly, staff participation rate (3%) was rarely reported, and none reported on staff representativeness. Level of expertise of the delivery agent included trained counselors, registered nurses, trained midwife, doulas, and social workers, lactation consultants and psychotherapists. The cost of adoption (i.e., start-up costs) was reported in one study and showed that the total cost of the intervention was US$3840 and US$301 / per mother.

### Implementation

The proportion of studies that reported across implementation indicators was 47% overall, but only 15% for core indicators. On average, indicators related to reach and implementation were the only factors that were reported with some regularity when considering all indicators associated with each dimension. However, across core RE-AIM indicators sample size and breastfeeding outcomes were reported in the vast majority of articles. Unfortunately, external validity indicators related to representativeness of participants, sites, or delivery staff were rarely reported. Similarly, adherence to intervention protocol, and indicator of internal validity, were only reported in a small proportion of articles. Intervention description was reported in 30 studies (97%), intervention duration in 27 studies (87%) and ranged from 2 weeks to 2 years. A statement of theory or principles used to develop the intervention was described in ten studies (32%) and included implementation of the Center Pregnancy Model, Theory of Planned Behavior and Social Cognitive Theory. Finally, cultural components were reported in 11 studies (35%) and included intervention adaptations based on focus group feedback from Black mothers, individualized educational curriculum, and community collaboration.

### Maintenance

The proportion of studies that reported across individual level maintenance indicators was 14% overall and 18% for core indicators. The overall proportion of studies that reported across organizational level maintenance indicators was 5% overall and 13% for core indicators. The degree to which participants were lost to follow-up was reported in six studies (19%) and five studies (16%) provided a description of individual behavior assessed at some duration following completion of the intervention. A description of program continuation after study completion was reported in four studies (12%).

## Discussion

The purpose of this paper was to determine the internal and external validity-related reporting of studies examining breastfeeding interventions among Black populations, using the RE-AIM framework. Overall, we documented that both internal validity-related indicators and external validity-related indicators are under-reported. External validity indicators related to representativeness of participants, sites, or staff were rarely reported. Similarly, adherence to intervention protocol, and indicator of internal validity, were reported in only a small proportion of articles. Still, findings related to effectiveness indicated that breastfeeding interventions increased breastfeeding rates. Our findings showed higher reporting of recruitment rates which provides insights on potential strategies that could be used for future intervention planning on Black populations, such as providing flyers at community-based locations (churches, libraries, prenatal clinics, community health centers), WIC agencies, and brochures thorough the target communities and at local health fairs.

While reviewing intervention studies among Black lactating women, it was found that cultural components of the breastfeeding interventions were not regularly reported. Concepts such as collectivism (familism, respect, kinship) and positive images of Black people breastfeeding need to be reported as they may play a strong role in influencing health behaviors (e.g., breastfeeding). It would also be helpful for future program planners to adopt evidence-based approaches that incorporate culture. Only including Black lactating persons without including cultural components is a missed opportunity to honor and acknowledge an instrumental identity [22]. Quality examples of strategies to advance breastfeeding rates and decrease racial disparities could be found at the Communities and Hospitals Advancing Maternity Practices (CHAMPS) program in Mississippi [23] and on initiatives such as the Black Girls Breastfeeding Club website [24] and Black Breastfeeding Week that provides culturally-informed breastfeeding resources and education to Black mothers and perinatal care providers.

Surprisingly, reporting of adoption had lower rates than what has been previously published [20]. Transparency is needed so that agencies can make the best decision for adopting an evidence-based, practical, and culturally informed intervention. Also reported at lower rates were all the measures of cost such as cost-effectiveness (i.e., save $ per life / per year), cost-adoption which is the price of adoption across all levels of the interventions, and also cost of implementation which is the ongoing cost of the intervention delivery across all levels. Furthermore, important components such as staff participation rate, setting representativeness and measures were less often reported. Disclosing how an intervention is supported (i.e., staff details) and intervention site information could be helpful in planning future interventions. Knowledge on components that relate to internal and external validity could be useful in better understanding and explaining breastfeeding initiation, continuation, and duration. Sustainability of the interventions was the RE-AIM dimension with lowest reporting with only one study describing how the intervention was integrated into the delivery system and information on ongoing cost.

From a methodological perspective, across the review we found that there was little difference in the evaluation of proportional reporting of core indicators, when compared to expanded indicators, of each RE-AIM dimension. This was consistent across all dimensions with the exception of implementation. It is noteworthy that the expanded indicators of implementation include a description of different aspects of the intervention such as duration, frequency, and content. An increased characterization of intervention components is critical for replication [25] and the underlying rationale for the expanded implementation indicators that were abstracted. However, we also demonstrated the need to evaluate the core implementation indicators that align with the treatment fidelity and cost. Without reporting implementation fidelity (which 80% of the studies did not), a lack of effectiveness due to type 3 error cannot be ruled out.

## Conclusion

The need for culturally-informed breastfeeding promotion programs that focus on addressing the needs and desires of Black families is clear. Evidence-based and culturally-informed strategies could be more readily incorporated into practice if consistent and detailed reporting on internal and external validity occurred. Future research would benefit from better reporting on individual level of representativeness; setting representativeness; cost effectiveness of the interventions; sustainability elements and consistency of reporting on fidelity and all measures of cost. Though breastfeeding is a biological norm, it is evident how important it is for the access and quality of culturally-congruent breastfeeding support, services, and resources to be available and affordable to ensure breastfeeding continuation.

## Supplementary Information

**Additional file 1.** The RE-AIM dimensions, definitions, and examples.

**Additional file 2.** Search strategies.

**Additional file 3.** Characteristics of studies included in systematic review [26–56].

## Data Availability

All data generated during this study and supplementary information are included in this published article.

## Abbreviations

CHAMPS: Communities and Hospitals Advancing Maternity Practices
PRISMA: Preferred Reporting Items for Systematic Reviews and Meta-Analyses
RE-AIM: Reach, effectiveness, adoption, implementation, and maintenance
US: United States
WIC: Women, Infants and Children
