# Supplementary material for: A systematic literature review of breastfeeding interventions among Black populations using the RE-AIM framework
Source: Int Breastfeed J. 2022 Dec 17;17:86. doi: 10.1186/s13006-022-00527-z (PMC9758845; doi:10.1186/s13006-022-00527-z)
Supplement: Supplementary file 1 — Additional file 1. The RE-AIM dimensions, definitions, and examples. [file 13006_2022_527_MOESM1_ESM.doc]

| Article ID Number | Located in the assignment sheet |
| --- | --- |
| Author, Year, Country | Self explanatory |
| Target Population | Brief description of the targeted population (older adults, pre-diabetic women) |
| Study Setting | The location(s) where the intervention is delivered.  Example: university/clinical/community/faith-based/home/worksite |
| Topic Area | List the health behavior or health topic that is covered (such as cancer, policy, diabetes…) |
| Purpose | What is the purpose of the manuscript; provide details that will give us highlights of the paper (e.g., the purpose of this paper was to report on the short term effectiveness of a dietary program, with special attention on the feasibility (adoption) of the program in a clinical setting). |
| Study Design | RCT, CC, Observational, etc. |
| Methods used: | Quantitative, qualitative, review article, meta analysis, narrative piece… |
| Level/Unit of Analysis: | Individual, setting, community, combination... |
| Companion Article | Is there a companion article to this particular intervention? (yes = 1; no =0) |
| Citation | If so, what is the citation we will need to search (later). |
| REAIM Overview Table * O= organizational I= individual | |
| Assessed? | The assessed column is simply a description of if this dimension is addressed (1 = yes; 0=no) |
| Change or Description or Neither | For this column indicate if the intervention attempted to change the outcome related to this dimension; described the outcome; or did neither |
| Measure | This is the specific information on how the outcome was assessed. |
| Challenge/Finding? | Description of any finding based on the study or if there were challenges recorded by the authors |
| Reach | The proportion & representativeness of individuals willing to participate in a given intervention |
| Described target population | A brief description of the broader target population (i.e., not simply of the study sample).  Example: The target population included all women within the community health center who were over the age of 18 and were not meeting the recommended guidelines for physical activity. |
| Demographic & behavioral information | Gender, age, educational attainment, occupation, SES, behavioral outcomes. |
| Method to identify target population | Describe the process by which the target population was identified for participation in the study.  Example: All patients who were part of the target population were identified using the electronic medical record. |
| Recruitment Strategies | Describe the methods used to recruit participants into the study. Example: We used a series of flyers; presentations; mass media; and word of mouth strategies to recruit participants. |
| Inclusion criteria | Explicit statement of characteristics of the target population that were used to determine if a potential participant is eligible to participate. Example: The inclusion criteria are… |
| Exclusion criteria | Explicit statement of characteristics that would prevent a potential participant from being eligible to participate. Also the percent excluded may be reported.  Example: The exclusion criteria are… |
| # Eligible and invited(exposed) to recruitment | The total number of eligible participants contacted for participation.  Example: 300 people were contacted for the study. After a screener was administered, it was found that of those 300 people contacted, 250 people were eligible. Therefore 250 is the denominator. |
| Sample size | The number of people who agree to participate (e.g. n= ) |
| Participation rate | Sample size divided by the target population denominator. .  Example: 200 (number of people agree to participate)/250 (number of eligible participants contacted for participation)=80% |
| # of Characteristics | Total number of comparisons made between target population and study sample with a description of what those comparisons were  Example: Participants were compared to non-participants for: activity level, gender, age... |
| # of statistically significant comparisons | Total number of statistically significant comparisons made between target population and study sample with a description of what those comparisons were.  Example: When compared to participants, non-participants were more likely to be older physically inactive females. |
| Cost of recruitment | The cost of recruitment can reflect monetary and/or time units.  Example: The overall cost of recruitment strategy A (flyers) was $1000 versus the overall cost of recruitment strategy B (newspaper advertisements) was $200. Could also be coded in cost per participant recruited. |
| Use of qualitative methods to measure reach | Reporting on non-quantitative aspects of reach. Observations in words, sentences, descriptions or codes. Some common methods include key informant interviews, focus groups, or even field notes that provide information on perceptions, feelings, opinions, experiences, etc. |
| Efficacy/Effectiveness | The influence of an intervention on important outcomes, including potential negative effects, quality of life, & economic outcomes |
| Report of Breastfeeding Outcomes | Variables that assess measures of breastfeeding (e.g., initiation, continuation, duration, exclusivity, knowledge, attitudes, intent) |
| Report of Mediators | Variables that explain the extent to which the particular variable accounts for the relationship between the predictor and the criterion. |
| Report of Moderators | List of variables: qualitative (e.g., sex, race, class) or quantitative (e.g., level of reward) that influence the direction and/or strength of the relationship between the treatment and the outcome. |
| Intent-to-treat or present at FU? | Intent to treat analysis:  when participants in trials are analyzed in the groups to which they were randomized, regardless of whether they received or adhered to the allocated intervention. Example, will typically use the term intent to treat or will describe an imputation that was used to account for missing data in the analysis.  Present at Follow-up analysis: when only participants who completed the follow-up assessment are included in the analysis of efficacy/effectiveness.  Example: Only those participants who completed both the baseline and follow-up measures were included in the analysis. |
| Imputation procedures (specify) | Substitution of some value for missing data.  Example: Multiple imputation methods were used to impute missing minutes of PA data at 3 months… |
| Quality of life measure | Includes a measure of quality of life with some latitude for coding articles that refer to well-being or satisfaction with life. |
| Measure unintended consequences (negative) and results | To evaluate unanticipated consequences and results that may be a product of the intervention and may have caused unintended harm.  Example: In a PA promotion program, female participants had an increased rate of injury. |
| Percent attrition (at program completion) | The proportion that was lost to follow-up or dropped out of the intervention. This is calculated by dividing the number of participants who did not complete the intervention by the number of participants who began the intervention.  Example: 100 participants began the intervention and 20 participants did not complete the intervention. So there was 20% attrition. |
| Cost effectiveness | Code as reported if specific mention and amounts are provided for the cost of the intervention.  Example: The new strategy would save $1,000 per life per year when compared to the current practice. |
| Use of qualitative methods to measure efficacy/effectiveness | Obtaining qualitative feedback from participants on the degree to which they felt the intervention was efficacious/effective. Some common methods include focus groups, interviews, diaries (text/pictures). |
| Adoption - Diffusion - Setting Level | The proportion & representativeness of locations willing to initiate & adopt an intervention |
| # eligible and invited(exposed) | Total sites that met eligibility criteria and were approached for intervention delivery. |
| # Participating | The total number of sites that agreed to participate. |
| Participation rate | The proportion of sites eligible and contacted that participated. |
| Description of targeted location | Characteristics that would be considered an ideal location for the intervention. |
| Inclusion/exclusion criteria of setting | The explicit statement of characteristics of the setting that were used to determine if a potential setting is eligible to participate.  Example: The inclusion/exclusion criteria are... |
| Description of intervention location | The explicit statement of characteristics of the location of the intervention.  Example: size of location; resources available staff information; number of eligible locations; work environment/climate |
| Method to identify setting | Describe the process by which the location was identified for participation in the study. |
| # of Comparisons | Total number and type of comparisons of targeted intervention sites and those that participated, including a list: size, location, etc. |
| # of statistically significant comparisons | Total statistically significant number and type of comparisons of targeted intervention sites and those that participated, including a list: Schools in urban areas were significantly less likely to agree to participate. |
| Average # of persons served per setting | Calculated average number of participants at each site. |
| Adoption - Diffusion - Staff Level | The proportion & representativeness intervention staff willing to initiate & adopt an intervention |
| # eligible and invited(exposed) | Total staff that met eligibility criteria and were approached for intervention delivery. |
| # Participating in delivery | The total staff members that agreed to participate. |
| Participation rate | The proportion of the staff that was eligible and contacted and participated. |
| Method to identify target delivery agent | Describe the process by which the target delivery agent was identified for participation in the study.  Example: All staff at the intervention location that had expertise in leading PA classes was identified by supervisors at the intervention location. |
| Level of expertise of delivery agent | Training or educational background in relevant area; Degrees, certifications of delivery agents (such as PhD, Masters, Registered Dietitian, etc.) |
| Inclusion/exclusion criteria of delivery agent | The explicit statement of characteristics of the delivery agents that were used to determine if a potential delivery agent is eligible to participate.  Example: The inclusion/exclusion criteria are... |
| # of Comparisons | Total number and type of comparisons of targeted staff members and those that participated, including a list: age, BMI, education |
| # of statistically significant comparisons | Total statistically significant number and type of comparisons of targeted intervention sites and those that participated, including a list: Staff members who were overweight were less likely to agree to deliver the program. |
| Measures of cost of adoption | The price of adoption across all levels of the intervention. At least some mention of start-up (i.e., **not ongoing** just **one time** (start-up)) costs. |
| Dissemination beyond originally planned | The spread of the intervention beyond what was planned before the start of the intervention |
| Use of qualitative methods to measure adoption | Used qualitative methods to understand the process of adoption.  Example: focus groups, interviews of adoption settings or delivery agents |
| Implementation | How consistently various elements of an intervention are delivered as intended by intervention staff, & the time & cost of the intervention |
| Theories | Explicit statement of theories or principles used to develop the intervention  Example: social cognitive theory, theory of planned behavior, SEM, D&I-based theories |
| Intervention number of contacts | Total number of encounters with participants. Could include face-to-face meetings, telephone calls, newsletters etc. |
| Timing of contacts | Describe when the intervention contacts occur over the course of the intervention.  Example: For the first month participants received one telephone call per week and in every month thereafter they received a call a month until the end of the 12 month intervention |
| Duration of contacts | Length of each intervention contact.  Example: The first 4 calls lasted about 20 minutes each, the other 11 lasted about 10 minutes each. |
| Extent protocol delivered as intended | Description of fidelity to the intervention protocol.  Example: a checklist of program components assessed by delivery agent(s) |
| Consistency of implementation across setting and delivery agents | Description of the degree of similarities between multiple settings sites & delivery agents |
| Participant attendance/completion rates | The proportion of the intervention that the participants received, on average.  Example: Participants attended 4 of the 6 meetings on average. |
| Measure of cost | The **ongoing** cost of delivery across all levels of the intervention |
| Use of qualitative methods to measure implementation | Used qualitative methods to understand the process of implementation.  Example: focus groups, interviews |
| Maintenance | The extent to which participants make & maintain a behavior change & the sustainability of a program or policy in the setting in which it was intervened |
| Was individual behavior assessed at some duration following the completion of the intervention? (give duration of follow-up) | Description of follow-up outcome measures of individuals available at some duration after intervention termination  Example: 6 months after the intervention ended participants had returned to baseline levels of PA. |
| Attrition | Describe the degree to which participants were lost to follow-up (and the reasons) during the period in time from the interventions completion to the follow-up. |
| Use of qualitative methods to measure individual maintenance | Used qualitative methods to understand the process of individual level maintenance of changes to the primary outcome.  Example: focus groups, interviews |
| Report alignment to  organization mission | Was the intervention designed to align with the delivery organization’s mission, values (explicit statement). |
| Is the program still in place? | Description of program continuation after completion of the research study. |
| If no: reason for discontinuation | Description of why the intervention was terminated |
| If yes: was the program modified? Specify | Description of any changes that were made to the original program |
| Was the program institutionalized? | Description of the how the intervention was integrated into the delivery system through methods such as policy changes, job description changes. |
| Attrition | Describe the degree to which sites were lost to follow-up (and the reasons) during the period in time from the interventions completion to the follow-up. |
| Use of qualitative methods to measure organizational level maintenance | Used qualitative methods to understand the process of intervention sustainability at the organizational level |
| Comparative Results | |
| Weight Measure | Yes or No |
| Disease self-management | Yes or No |
| Quality of Life Measures | Yes or No |
| Physical Activity | Yes or No |
| Dietary | Yes or No |
| Smoking/Substance | Yes or No |
| Diabetes  Gestational Diabetes  Gestational Hypertension  C-Section  Complications During Pregnancy  Complications During Labor  Complications During Birth Complications During Postpartum | Yes or No  Yes or No  Yes or No  Yes or No  Yes or No  Yes or No  Yes or No  Yes or No |
| Other? | Yes- Please provide |

This file was adapted from the RE-AIM Coding Companion Guide available on https://www.re-aim.org/resources-and-tools/measures-and-checklists/
