# Supplementary material for: A systematic literature review of breastfeeding interventions among Black populations using the RE-AIM framework
Source: Int Breastfeed J. 2022 Dec 17;17:86. doi: 10.1186/s13006-022-00527-z (PMC9758845; doi:10.1186/s13006-022-00527-z)
Supplement: Supplementary file 2 — Additional file 2. Search strategies. [file 13006_2022_527_MOESM2_ESM.docx]

Additional File 2: Search Strategies.

Search Strategies

(breastfeeding[MeSH]) OR (breastfeed[MeSH]) OR (“breastfeeding intervention”[MeSH]) or (breast feeding[MeSH]) AND (African American[MeSH])

(breastfeeding[tiab]) OR (breastfeed[tiab]) OR (“breastfeeding intervention”[tiab]) or (breast feeding[tiab]) AND (African American[tiab])
