# Supplementary material for: A systematic literature review of breastfeeding interventions among Black populations using the RE-AIM framework
Source: Int Breastfeed J. 2022 Dec 17;17:86. doi: 10.1186/s13006-022-00527-z (PMC9758845; doi:10.1186/s13006-022-00527-z)
Supplement: Supplementary file 3 — Additional file 3. Characteristics of studies included in systematic review [26–56]. [file 13006_2022_527_MOESM3_ESM.docx]

| **Author** | **Year** | **Target Population** | **Study Setting** | **Study Design** | **Methods Used** | **Level/ Unit Analysis** | **Cultural Components** | **Results** |
| --- | --- | --- | --- | --- | --- | --- | --- | --- |
| Berry et al. [26] | 2013 | Women who are diagnosed with gestational diabetes mellitus (GDM) | University of North Carolina at Chapel Hill (UNC-CH) Schools of Nursing and Medicine and two clinical sites, Rex Healthcare and WakeMed | RCT | Quantitative | Group | N/A | Protocol; Does not have outcome results |
| **Rozga et al. [27]** | 2015 | Low-income women enrolled prenatal in Michigan’s Breastfeeding  Initiative Peer Counseling Program. | Home/hospital | Secondary analysis | Quantitative | Group | N/A | For each additional home, phone and other (peer counselling) PC contact there was a **significant reduction in the hazard of discontinuing any breastfeeding by 6 months** (hazard ratio (HR)=0.90 (95 % CI 0.88, 0.92); HR=0·89 (95 % CI 0.87, 0.90); and HR=0.93 (95 % CI 0.90, 0.96), respectively) and **exclusive breastfeeding by 3 months** (HR=0.92 (95 % CI 0.89, 0.95); HR=0·90 (95 % CI 0.88, 0.91); and HR=0.93 (95 % CI 0.89, 0.97), respectively) |
| Wasser et al. [28] | 2017 | African American pregnant women and families living in central North Carolina | Home visitation | RCT | Quantitative | Individual | N/A | Protocol. Does not have outcome results |
| Leruth et al. [29] | 2017 | African American families and women disproportionately less likely to nurse | WHS program site in Chicago, Illinois or the participant’s home | Descriptive | Quantitative and qualitative | Community | N/A | Breastfeeding duration at 6 months: non-significant; reported percentages |
| **Arlotti et al. [30]** | 1998 | Low-income women (African American, Hispanic, White) | The WIC department of a County Public Health Unit, located in a medium-sized North Florida city, | Longitudinal | Qualitative and quantitative | Combination | N/A | Attending a breastfeeding class was significantly associated with **increased duration of exclusive breastfeeding**, **t(34) = 2.16,~ < 04.** Specifically, attendance at a breastfeeding class was associated with an increase of 3.14 weeks in duration of exclusive breastfeeding. When group membership (counselor vs. no counselor) was added to a model containing the aforementioned predictors, it was significantly related to **duration of exclusive breastfeeding**, **t(34) = 2.01, p < .05** |
| Bonuck et al. [31] | 2005 | Hispanic and/or Black women | Two community health centers serving low income | RCT | Quantitative | Community | All study materials were translated professionally into Spanish. These translations were checked for cultural concordance with local dialects by native Spanish-speaking study staff members. Practice with a culturally appropriate lactation doll and nipple was offered. | Breastfeeding intensity: non-significant increases; reported confidence intervals |
| Caulfiedl et al. [32] | 1998 | Black women at prenatal care clinics who are WIC eligible | WIC clinics | RCT | Quantitative | Individual | N/A | Breastfeeding initiation: non-significant increases; reported percentages |
| Celi et al. [33] | 2005 | Pregnant women participating in Project Viva | Offices of a multisite multispecialty group practice in eastern Massachusetts | Cohort | Quantitative | Individual | N/A | Breastfeeding initiation: non-significant increases; reported confidence intervals |
| **Edwards et al. [34]** | 2013 | African American and others | Home | RCT | Quantitative | Individual | African American Doulas from surrounding communities who had not been trained as health professionals were assigned to the participants. These Doulas shared their own breastfeeding experiences or the breastfeeding experiences of others from within their community | **Mothers who received doula services were more likely to breastfeed p=.04** |
| Furman et al. [35] | 2016 | African American Women who were breastfeeding | Home | Community-based participatory research | Quantitative | Individual | Authors V.D., S.K., and L.F. created a draft intervention with input from focus groups that included the target population | Any breastfeeding and exclusive breastfeeding: Non-significant increases; reported confidence intervals |
| Grady and Bloom [36] | 2004 | Pregnant adolescents and teens 19 y/o and younger | Barnes Jewish Hospital in St.  Louis, Missouri | Descriptive | Quantitative | Community | N/A | Breastfeeding rates: non-significant increases; reported percentages |
| Gross et al. [37] | 1998 | Black women at prenatal care clinics who are WIC eligible | WIC clinics | RCT | Quantitative | Individual | Ethnographic research was conducted in the WIC clinics to help design the interventions and develop the survey instruments. | Breastfeeding at 8 and 16 weeks and Rate of decline in breastfeeding at 8 and 16 weeks postpartum: non-significant differences; reported percentages |
| **Howell et al. [38]** | 2014 | Latina and Black | Hospital located in New York City | Effectiveness | Quantitative | Individual | The intervention was revised based on feedback from focus groups which included Black and Latina mothers. All of the intervention materials were also translated into Spanish. | **Breastfeeding duration p=.02**  **Less likely to quit breastfeeding over 1^st^ 6 months after delivery (hazard ratio, 0.79; 95% confidence interval, 0.65–0.97)** |
| Ickovics et al. [39] | 2015 | Pregnant women aged 14 to 21 years old, less than 24 weeks of gestation | Clinical | RCT | Quantitative | Individual | N/A | Breastfeeding initiation: non-significant increases; reported percentages |
| **Ickovics et al. [40]** | 2007 | Pregnant women aged 14-25 years old | University-affiliated hospital prenatal clinics | RCT | Quantitative | Group | N/A | **Breastfeeding initiation p<.05** |
| **Thomson at al. [41]** | 2014 | Mothers and their infants residing in the rural Mississippi Delta region of the US | Home-based | RCT | Quantitative | Group | Materials are tailored to the age of the child and responsive to parental information requests. Parents as Teachers Enhanced delivered to participants randomized to the experimental arm, builds upon the MIECHV program, PaT, by adding culturally tailored, maternal weight management and early childhood obesity prevention component Elements based upon DPP principles include a flexible, culturally sensitive, individualized educational curriculum taught on a one-to-one basis. | **Breastfeeding knowledge scores: p<.05**  **Breastfeeding intent and beliefs as well as pre-pregnancy weight class significantly predicted breastfeeding initiation**  **p<.05** |
| Kellams et al. [42] | 2015 | Low-income minority populations | Prenatal care office visit | RCT | Quantitative | Individual | N/A | Breastfeeding initiation and duration: non-significant differences |
| **Kistin et al. [43]** | 1990 | Black mothers | Cook County Hospital Midwife Prenatal Clinic | RCT | Quantitative | Community | N/A | **Breastfeeding rates: p<.05** |
| Lewkowitz et al. [44] | 2018 | Socioeconomically disadvantaged (SED) African American women with overweight and obesity. | University-based hospital/home | RCT | Quantitative | Combination | N/A | Breastfeeding initiation: non-significant differences; reported confidence intervals |
| Meier et al. [45] | 2004 | Mothers with Very-low-birth-weight-infants who were able to provide their own milk to their babies | Hospital | Retrospective | Quantitative | Combination | The breastfeeding peer counselors attend weekly Mothers’ Milk Club luncheons with their infants and serve as breastfeeding experts and role models for other women. Additionally, the breastfeeding peer counselors make telephone calls to the mothers after their infants are discharged from the NICU and can access the Mothers’ Milk Club Taxi for home visits, as necessary. | Breastfeeding initiation: non-significant differences |
| **Merewood et al. [46]** | 2019 | African American Infants | Clinical | Effectiveness | Quantitative | Combination | Given the background of racial inequities in Mississippi, direct and meaningful collaboration with the community was critical to prepare women for changing practices and to support women who were breastfeeding postpartum in regions with low breastfeeding rates | **Breastfeeding initiation and exclusivity: p< .05** |
| Moon et al. [47] | 2017 | African American Mothers and Infants | N/a | RCT | Quantitative | Individual | N/A | Breastfeeding duration: non-significant differences |
| Pugh et al. [48] | 2010 | low-income women | University hospital and community hospital | RCT | Quantitative | combination | N/A | Breastfeeding duration: non-significant differences |
| Pugh et al. [49] | 2002 | Low-Income Breastfeeding Women | Hospital/home | RCT | Quantitative | Individual | N/A | Breastfeeding duration: non-significant differences |
| **Reno [50]** | 2018 | Low-income African American women | Prenatal care clinic, a community-based perinatal program, neighborhood childcare center, and a local library. | Quasi-experimental | Quantitative | Individual | Dynamic breastfeeding assessment process (D-BAP) developed with input from (design/development) low-income African American women; resilience (facilitate, supportive, empower women); barriers to breastfeeding; decenter the researcher; culturally responsive intervention; mixed methods feedback on content and experience of D-BAP; community-based certified lactation counselor; worksheet and breastfeeding handouts developed for Black women | **Breastfeeding self-efficacy: p<.04** |
| Ross-Cowdery et al. [51] | 2017 | Women seeking prenatal care | Hospital | Quasi-experimental | Quantitative | Individual | N/A | Breastfeeding knowledge scores and intention: non-significant differences |
| Sisk et al. [52] | 2006 | Mothers of very low birth weight infants | Hospital/NICU | Quasi-experimental | quantitative | Individual | N/A | Breastfeeding intention: non-significant differences |
| Spinelli et al. [53] | 2013 | Black women with major depression | Hospital | RCT | Quantitative | Group | N/A | Protocol; Does not have outcome results |
| **Stuebe et al. [54]** | 2016 | Pregnant women with gestational diabetes mellitus | Clinic | RCT | Quantitative | individual | N/A | **Less likely to stop breastfeeding (adjusted HR 0.40, 95% CI 0.21–0.74) or to introduce formula (adjusted HR 0.50, 95% CI 0.34–0.72)** |
| Thomson et al. [55] | 2017 | African American women | Home | RCT | Quantitative | Individual | Curriculum: flexible, culturally sensitive, individualized; Parent Educator: African American, college educated women residing in the target communities; collected data and delivered content | Protocol; Does not have outcome results |
| **Wambach et al. [56]** | 2011 | Adolescent mothers | Prenatal clinics  departments, affiliated with three urban public and private teaching hospitals; most sites participated in WIC and now were designated as Baby Friendly | RCT | Quantitative | individual | N/A | **Breastfeeding duration: p<.001**; breastfeeding initiation and exclusivity: non-significant differences |

Note: Bolded items reflect statistically significant breastfeeding outcomes that were reported.
